# A comprehensive assessment of RNA-seq protocols for degraded and low-quantity samples

**DOI:** 10.1186/s12864-017-3827-y

**Published:** 2017-06-05

**Authors:** Sven Schuierer, Walter Carbone, Judith Knehr, Virginie Petitjean, Anita Fernandez, Marc Sultan, Guglielmo Roma

**Affiliations:** Novartis Institutes for Biomedical Research, Novartis Pharma AG, Basel, Switzerland

**Keywords:** RNA-sequencing, Expression profiling, Benchmarking, Low quality, Low quantity, Differential expression

## Abstract

**Background:**

RNA-sequencing (RNA-seq) has emerged as one of the most sensitive tool for gene expression analysis. Among the library preparation methods available, the standard poly(A) + enrichment provides a comprehensive, detailed, and accurate view of polyadenylated RNAs. However, on samples of suboptimal quality ribosomal RNA depletion and exon capture methods have recently been reported as better alternatives.

**Methods:**

We compared for the first time three commercial Illumina library preparation kits (TruSeq Stranded mRNA, TruSeq Ribo-Zero rRNA Removal, and TruSeq RNA Access) as representatives of these three different approaches using well-established human reference RNA samples from the MAQC/SEQC consortium on a wide range of input amounts (from 100 ng down to 1 ng) and degradation levels (intact, degraded, and highly degraded).

**Results:**

We assessed the accuracy of the generated expression values by comparison to gold standard TaqMan qPCR measurements and gained unprecedented insight into the limits of applicability in terms of input quantity and sample quality of each protocol. We found that each protocol generates highly reproducible results (*R* ^2^ > 0.92) on intact RNA samples down to input amounts of 10 ng. For degraded RNA samples, Ribo-Zero showed clear performance advantages over the other two protocols as it generated more accurate and better reproducible gene expression results even at very low input amounts such as 1 ng and 2 ng. For highly degraded RNA samples, RNA Access performed best generating reliable data down to 5 ng input.

**Conclusions:**

We found that the ribosomal RNA depletion protocol from Illumina works very well at amounts far below recommendation and over a good range of intact and degraded material. We also infer that the exome-capture protocol (RNA Access, Illumina) performs better than other methods on highly degraded and low amount samples.

**Electronic supplementary material:**

The online version of this article (doi:10.1186/s12864-017-3827-y) contains supplementary material, which is available to authorized users.

## Background

In recent years, high-throughput RNA sequencing (RNA-seq) has become the method of choice to accurately probe the transcriptome of any biological specimens [1–6]. This method quantifies the expression levels of thousands of RNA transcripts within a single assay, while simultaneously allowing unbiased discovery of splicing variants [7], rare and novel transcripts [8], non-coding RNAs [9–12], and nucleotide changes [13]. In discovery settings RNA-seq replaced the use of microarrays [14, 15] to study human diseases [16, 17] or to identify novel drug targets [18–20], biomarkers [21], and compound mechanisms of action [22]. More recently, RNA-seq is transitioning from a discovery to a diagnostic tool with clinical utility in patient stratification, diagnosis, and individualized treatment [23]. However, working with human tissue specimens available from centralised biobanks, hospitals, research centres, or universities, can often result in poor quality and low yields of RNA due to pre-analytical factors (e.g. sampling methods, preservation conditions, storage conditions, and time) that can ultimately affect gene expression analysis.

Several next generation sequencing protocols are currently available for the profiling of RNA samples, each with its own strengths and weaknesses. These methods use different strategies to reduce the representation of abundant ribosomal RNAs (rRNA) in RNA-seq libraries prior to sequencing. Poly(A) + enrichment using oligo-dT coated beads is the most common approach to quantify the polyadenylated RNA fraction of the transcriptome including coding mRNAs. However, this method fails at profiling other RNA populations (e.g. non-coding RNAs) and suffers from biases when applied to low quality or low quantity RNA samples [24–26]. Ribosomal RNA depletion methods are better suited for the sequencing of RNA samples with lower quality since they reduce the highly abundant ribosomal RNAs from the total RNA samples using capture probes and offer an attractive option for the simultaneous detection of coding and non-coding RNAs [27]. Finally, RNA capture is a novel approach used to profile poor quality RNA samples like those extracted from formalin-fixed, paraffin-embedded (FFPE) tissue samples [28]; this method uses capture probes targeting known exons to enrich for coding RNAs. The “TruSeq” Stranded mRNA Kit, the “Ribo-Zero” rRNA Removal Kit, and the “RNA Access” Library Prep Kit represent respectively implementations of the poly(A) + enrichment, ribosome depletion and exome-capture approaches. These kits are commercial products (Illumina) with standardized, reproducible and easy to implement protocol steps and therefore suitable for any research laboratories conducting gene expression studies.

Numerous recent studies have compared different RNA-seq library preparation protocols. Some of these focused on degraded input RNA [24, 25, 28, 29], some others on low input RNA [10, 25, 30–32], or on general characteristics of the protocols [27]. For low input amounts (<100 ng), only protocols including a whole transcriptome amplification (WTA) step, such as NuGEN’s Ovation or Clonetech’s SMARTer [33], have been investigated. These protocols rely on additional PCR steps that are known to introduce amplification biases in the gene expression data [25, 30, 31]. A simultaneous assessment of both low and degraded input has been so far performed only by *Adiconis* et al. [25]; however, these authors considered samples with simultaneous low and degraded input only for one protocol and a single input amount (NuGEN’s Ovation protocol at 1 ng) [31].

Despite these comparative efforts and in the light of new protocol developments, there are fundamental technical questions that remain still unanswered. For instance, how does the newly available RNA Access protocol perform on degraded samples? And, does it provide any advantages over ribosomal RNA depletion methods? Furthermore, how does the performance of these different approaches change when lowering the input RNA quantity even below the recommended amounts? These questions become especially relevant in the clinical context where, for instance, processing of human biopsies often results in low amount and very heterogeneous quality RNA samples.

Here, we designed a study to evaluate the performance of the TruSeq, Ribo-Zero, and RNA Access library preparation kits on human reference RNA samples from the Microarray/Sequencing Quality Control consortium (MAQC/SEQC) [34] over a wide range of total RNA input amounts (from 100 ng down to 1 ng) and across three degradation stages (from intact to highly degraded). To our knowledge this is the first analysis which compares the recently commercialized RNA Access with the well-established TruSeq and Ribo-Zero protocols on the largest sample set of low quantity and low quality RNA samples ever investigated so far. Moreover, we went beyond the recommended amounts of each protocol to determine the minimum RNA quantities that can still deliver accurate gene expression results. Finally, we took advantage of the TaqMan qPCR values of about 1000 genes available for the SEQC samples to compare the three RNA-seq protocols to an orthogonal gold-standard. Based on our results, we provide the scientific community with a guidance on which protocol to use in relation to the quantity and quality of their RNA samples.

## Results

**Fig. 1:**
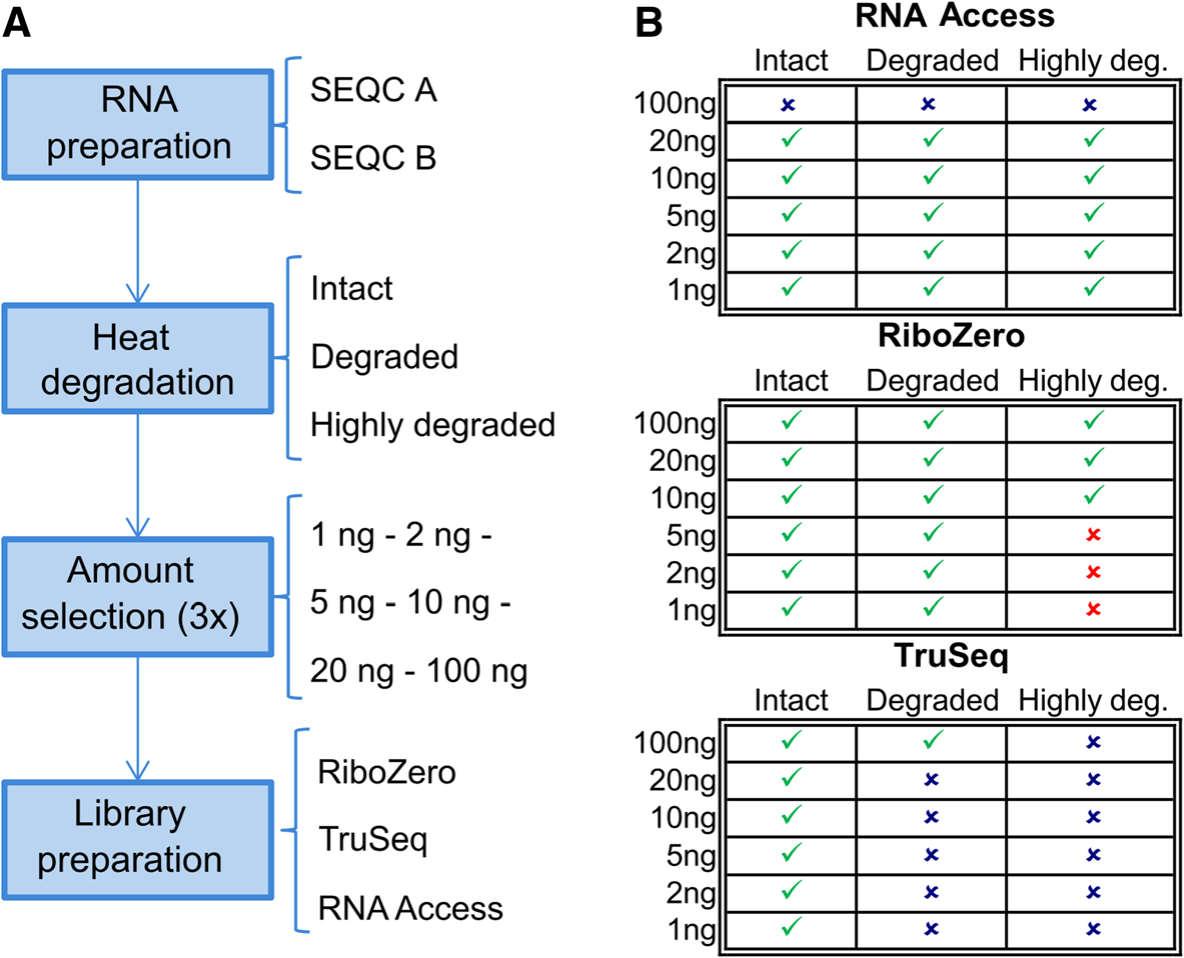
Generation of sequencing libraries and experimental design. **a** Schematic representation of the workflow to generate the sequencing libraries. RNA of the SEQC samples A and B is heat degraded to obtain three distinct RNA input qualities. Several input amounts between 1 ng and 100 ng are selected from the degraded RNA in triplicate and used for the library preparation with one of the three protocols (TruSeq, Ribo-Zero, or RNA Access). **b** Overview of the combinations of degradation stage, input amount, and library preparation protocol considered in this study. A green tick indicates a combination that was sequenced, a blue cross indicates a combination for which we decided not to generate a library either because the input amount was higher than the maximum recommended input amount (for RNA Access) or because previously published studies suggested inferior performance on degraded samples (for TruSeq), and a red cross indicates a combination for which no library was generated because libraries with higher input amounts already performed poorly

To evaluate the performance of RNA-seq methods in profiling non-optimal samples, we conducted a technical assessment of the three different RNA library preparation protocols mentioned above, namely TruSeq, Ribo-Zero and RNA Access, on two human reference RNA samples previously used in the MAQC/SEQC studies; these samples are the Universal Human Reference RNA (UHRR or SEQC-A) and the Human Brain Reference RNA (HBRR or SEQC-B) [34]. Figure 1a shows a schematic of the workflow and the different input choices considered at each step.

Overall we prepared a total of 222 sequencing libraries from 37 different combinations of degradation stage, input amount, and library preparation protocol each of which was applied to both samples in triplicate. Figure 1b gives an overview of the conditions selected in the study. The RNA libraries were sequenced on an Illumina HiSeq2500 in paired-end mode to a length of 76 bp ×2 generating a total of 3242 M reads. The sequencing depth of the different libraries ranged from ~19 M to ~95 M reads with an average of 49 M for the RNA Access samples, 39 M for the Ribo-Zero samples, and 43 M for the TruSeq samples. We used this comprehensive data set to benchmark the RNA-seq library preparation protocols on their efficiency to generate high-quality reads and consistent alignment rates, their ability to cover full-length annotated transcripts, their specificity in profiling protein-coding and non-coding RNAs, their accuracy in detecting gene expression changes by comparing them to TaqMan data, and their reproducibility and similarity by measuring the correlation of log fold changes within the same protocol at different input amounts and degradation levels as well as between the different protocols.

### Alignment statistics

**Fig. 2:**
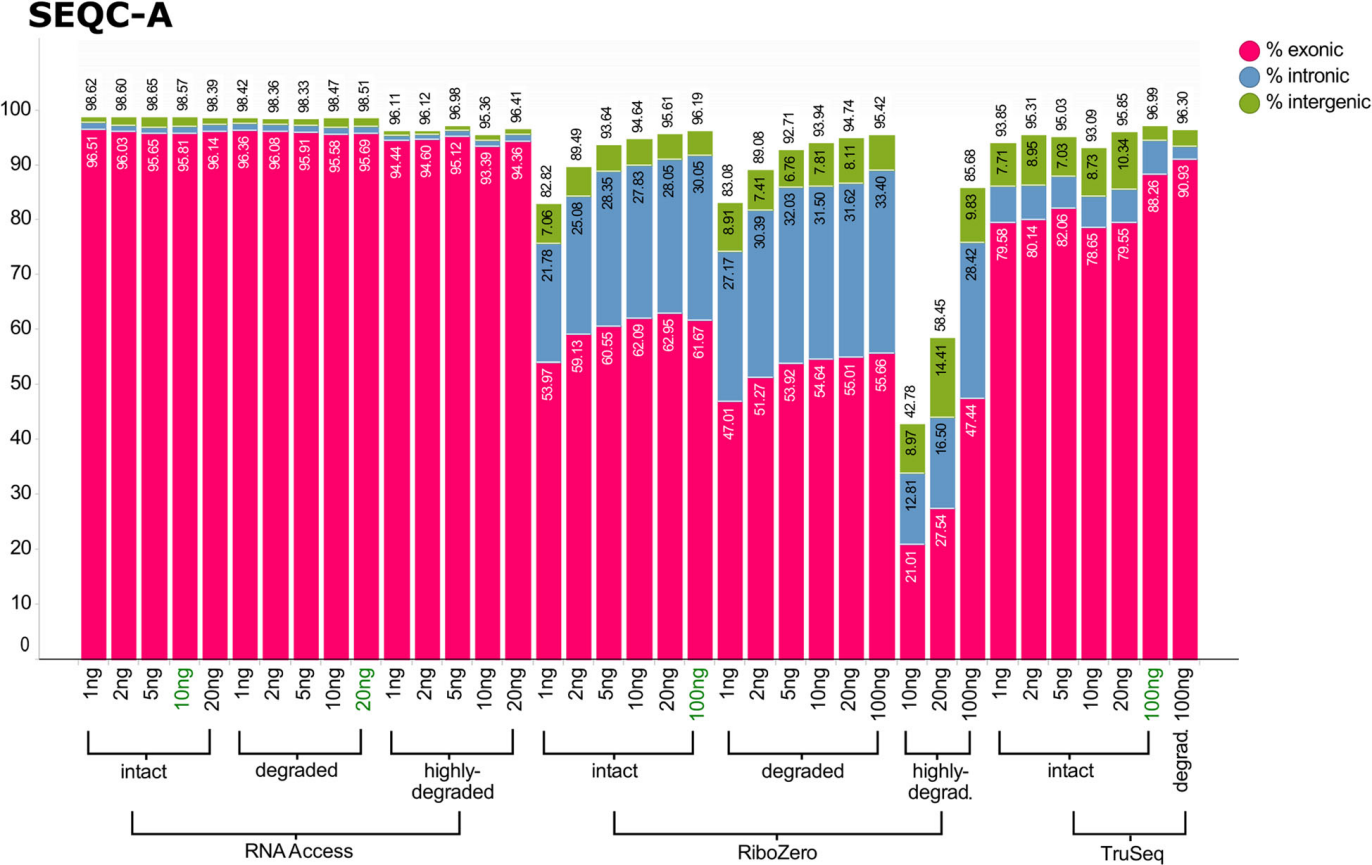
Bargraph of the alignment statistics for the SEQC-A sample and all three protocols. Each bar represents the averaged values across the three technical replicates per condition. The percentage of total aligned reads is represented by the height of the bar, and the percentage of reads aligning to exons is in red, introns in blue, and intergenic regions in green. The alignment statistics graph for the SEQC-B sample can be found in Additional file 1: Figure S4

We initially examined the overall alignment rates to the human genome (Fig. 2 and Additional file 1: Figure S4). Our results confirmed that the three library preparation protocols perform equally well on intact input RNA at the amounts recommended by the manufacturer (100 ng for TruSeq and Ribo-Zero, and 10 ng of intact RNA or 20 ng of degraded RNA for RNA Access). This is indicated by the high alignment rates ranging from ~96% to ~98.5% for all three approaches. However, the protocols behaved differently when the input amount was reduced or the sample quality decreased. For intact RNA, the alignment rate of RNA Access remained largely constant across all input amounts whereas we found a loss of about 3–4% aligned reads for TruSeq and about 10–15% aligned reads for Ribo-Zero with decreasing the input amounts down to 1 ng. When we considered the sample quality, the alignment results for degraded samples were comparable to the results of the intact samples for all three protocols. This is in agreement with previous studies [28]. However, for the highly degraded samples, the picture changed considerably. Whereas with RNA Access only a slight decrease of 2–4% aligned reads was observed even at the lowest input amount (1 ng) for SEQC-A (Fig. 2) and SEQC-B (Additional file 1: Fig. S4) respectively, we found a substantial drop of mapped reads (e.g. a decrease of 51% for SEQC-A and 72% for SEQC-B) at input amounts of 10 ng and 20 ng of highly degraded RNA input processed with the Ribo-Zero kit. Based on this poor performance, no further libraries were generated for lower input amounts and this degradation level with Ribo-Zero.

In addition, the protocols showed marked differences in the percent of reads aligned to exons, introns, and intergenic regions. For RNA Access the percentages of intronic and intergenic reads were both ~1% across all input amounts and sample quality categories, thus indicating high efficiency of the exome pull down by the capture approach. For TruSeq the percentage of intronic reads decreased with the sample quality from ~6% for intact samples to 2–3% for degraded samples, whereas the percentage of intergenic reads increased from ~2–3% for 100 ng input to ~6–10% for 20 ng and less. For Ribo-Zero the proportion of reads aligned to exons was considerably lower and dependent on the sample as previously reported (10). As expected with a total RNA sequencing approach, we observed between half to two thirds of the reads mapping to exons while the rest mapped to mostly intronic regions (30–34%) and to some extent to intergenic regions in intact and degraded samples. For highly degraded samples, the percentage of reads mapping to exons, introns, or intergenic regions was much more variable across technical replicates and input amounts; for SEQC-A the percentage was similar to the intact and degraded sample qualities but for SEQC-B up to 80% of the reads were mapped to intergenic regions.

All together these results indicate a consistent mapping performance of the RNA Access approach throughout all input amounts and degradation states, while the Ribo-Zero approach performed less well on highly degraded samples and at very low input amounts.

### Transcript coverage

**Fig. 3:**
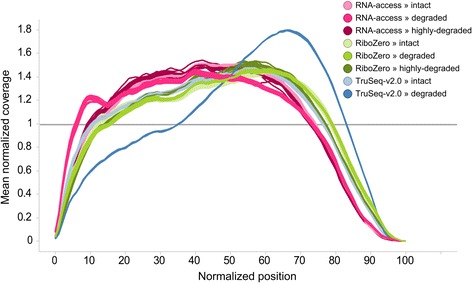
Normalized transcript coverage plot. Plot of the normalized average coverage of the 1000 most expressed transcripts for each sample condition as created by Picard

We next measured the variation in 5′ to 3′ coverage along each transcript (Fig. 3). Overall we observed similar and uniform transcript coverages for RNA Access and Ribo-Zero, independent of the degradation stage of the sample. In contrast, the TruSeq protocol had a marked difference between intact and degraded RNA, where the latter showed a strong 3′ bias; in other words, the proportion of sequences mapping to the 3′ regions of transcripts was largely increased compared to the 5′ regions. This bias is due to the 3′ oligo(dT)-dependent selection used in the Poly(A) + approach and underlines the limited use of this protocol on degraded RNA or in general on samples of heterogeneous quality. In contrast, the RNA Access method showed a slight 5′ bias. The consistency of the coverage profiles across the different degradation stages suggests that the Ribo-Zero and RNA Access protocols are better suited for the profiling of degraded or heterogeneous RNA sample populations than the standard TruSeq method.

### Gene level comparison

**Fig. 4:**
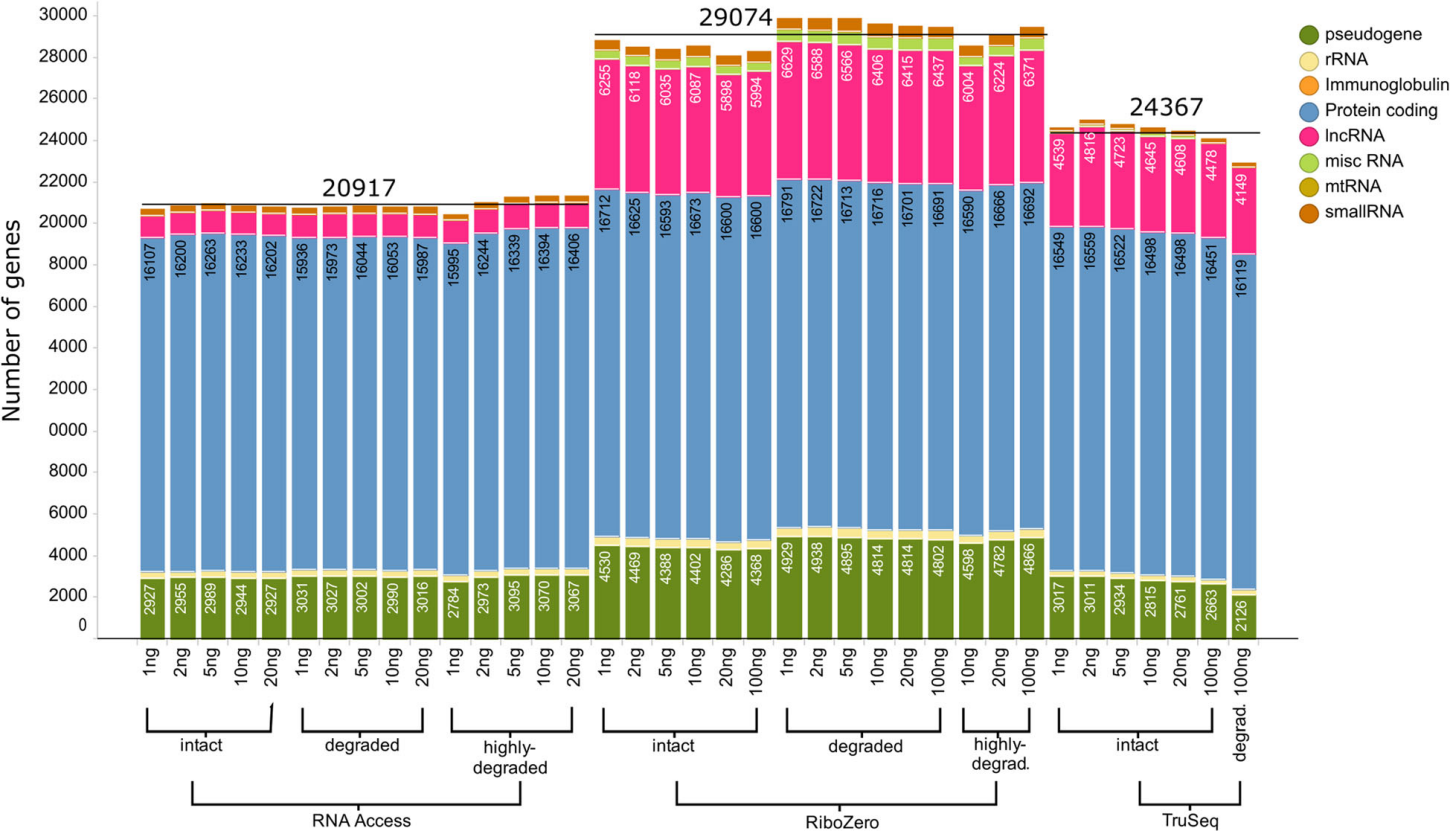
Bargraph of the number of detected genes across different protocols, degradation stages, and input amounts. The bar segments with the number of detected genes are listed by simplified Ensembl “Gene type” categories and the average number of detected genes per protocol is indicated by a black line. A gene is considered “expressed” if it has a FPKM value of at least 0.3 in one of the three technical replicates of at least one of the two samples (SEQC-A or SEQC-B)

We first investigated which annotated genes are detected by the three different protocols (Fig. 4), including protein coding genes, non-coding RNAs, and pseudogenes. To this purpose, we considered an arbitrary cutoff of 0.3 FPKM to call a gene ‘expressed’. Overall, RNA Access detected the least number of gene with an average of 20,917 expressed genes, followed by TruSeq with 24,367, and Ribo-Zero with 29,074. The number of detected genes was largely independent of the input amount and the sample quality. RNA Access showed a minor reduction in the number of detected genes at the lowest input amount of 1 ng; similarly, Ribo-Zero also showed a minor reduction at lower input amounts for highly degraded samples. The overall differences were reflecting the nature of the different protocols: Total RNA sequencing with Ribo-Zero could detect the largest transcript population, TruSeq identified most of the polyadenylated transcripts (including mRNAs and some non-coding RNAs) and RNA Access captured RNAs that are targeted by the design of its probes. Protein coding genes are clinically the most relevant and most studied category. Among protein coding genes, RNA Access detected an average of about 16.2 k genes which represents 97% of the 16.7 k protein coding genes detected by Ribo-Zero. The number of detected genes for TruSeq is in between with an average of 16.5 k. In all the other categories, the unbiased Ribo-Zero approach was more sensitive than the targeted protocols TruSeq and RNA Access. For example, a higher number of pseudogenes was detected with Ribo-Zero with an average of 4.7 k genes over 3.0 k for RNA Access and 2.8 k for TruSeq; Ribo-Zero is by far the best protocol to profile long non-coding RNAs (lncRNAs) as reflected by the high number of detected transcripts: on average 6.3 k lncRNAs for Ribo-Zero, 1.1 k for RNA Access and 4.6 k for TruSeq. These three categories covered over 95% of all detected genes for all three protocols. For the remaining categories, Ribo-Zero could detect 41% and 63% more small RNAs than RNA Access and TruSeq respectively, as well as an average of 506 miscRNAs against 113 for TruSeq and 44 for RNA Access.

**Fig. 5:**
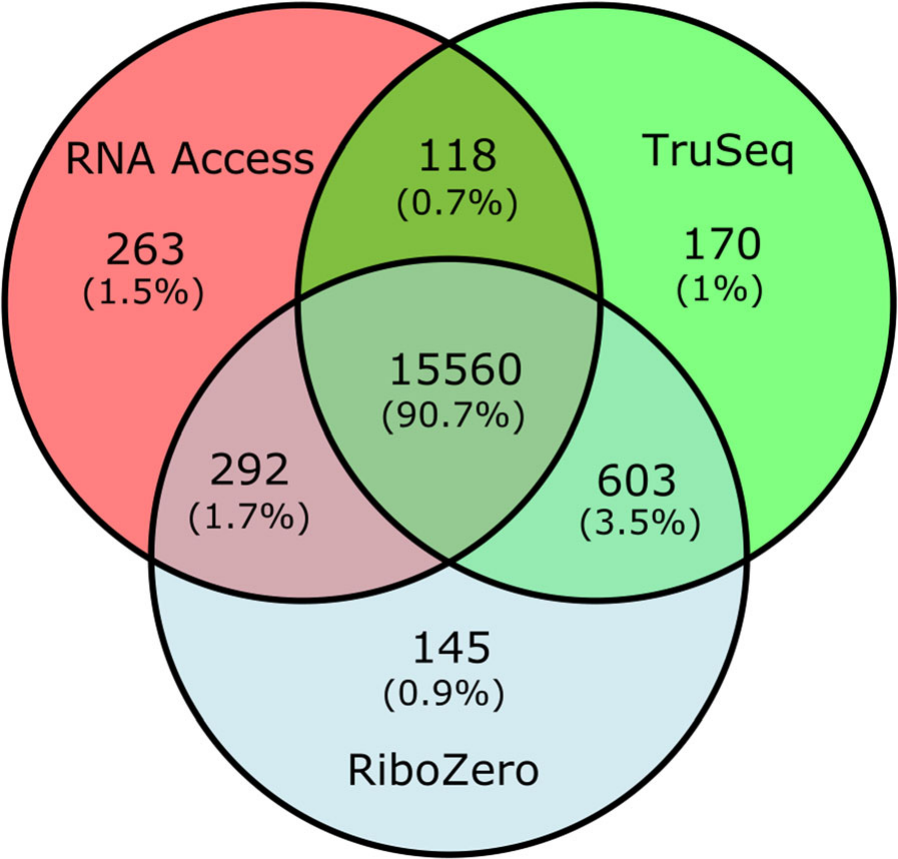
Venn diagram of the protein coding genes detected by each of the three protocols. Venn diagram of the protein coding genes detected by each of the three protocols on intact samples at the recommended input amounts (10 ng for RNA Access and 100 ng for Ribo-Zero and TruSeq). A gene is considered “expressed” if it has a FPKM value of at least 0.3 in one of the three technical replicates of at least one of the two samples (SEQC-A or SEQC-B)

We further investigated the overlap of detected genes between the different protocols. Due to the very heterogeneous characteristics of the different protocols on other gene categories, we focused this comparison on the protein coding genes and compared them with the recommend input amounts for intact RNA (10 ng of intact RNA for RNA Access and 100 ng for Ribo-Zero and TruSeq, Fig. 5). The vast majority of the protein coding genes (90.7%) was detected by all three protocols, while a small percentage of protein coding genes (0.9–1.5%) was specific to each protocol. In addition, the exclusive overlap between TruSeq and Ribo-Zero (3.5%) was slightly larger than the exclusive overlap between RNA Access and TruSeq (0.7%) or RNA Access and Ribo-Zero (1.7%). When we considered degraded samples with the same input amounts, we observed a very similar distribution of the overlap of detected genes between the different protocols (Additional file 1: Figure S5).

### Comparison to TaqMan qPCR data

To assess the accuracy of each protocol in detecting differential expression, we used publicly available qPCR data obtained for 1000 genes on SEQC-A and SEQC-B samples and generated by the MAQC consortium (12) as a gold standard reference. To this purpose, we compared the log fold-change values of the SEQC samples generated with the three RNA-seq methods to the TaqMan log fold changes since fold changes – as opposed to the direct comparison of absolute expression values – are a crucial parameter in the analysis of differentially expressed genes. In a differential expression analysis fold changes are usually accompanied by a measure of significance, such as a *p*-value, to capture the biological variability. However, in this study we did not include *p*-values in our assessment since the SEQC RNA-seq data consist only of technical replicates with no biological variability.

**Fig. 6:**
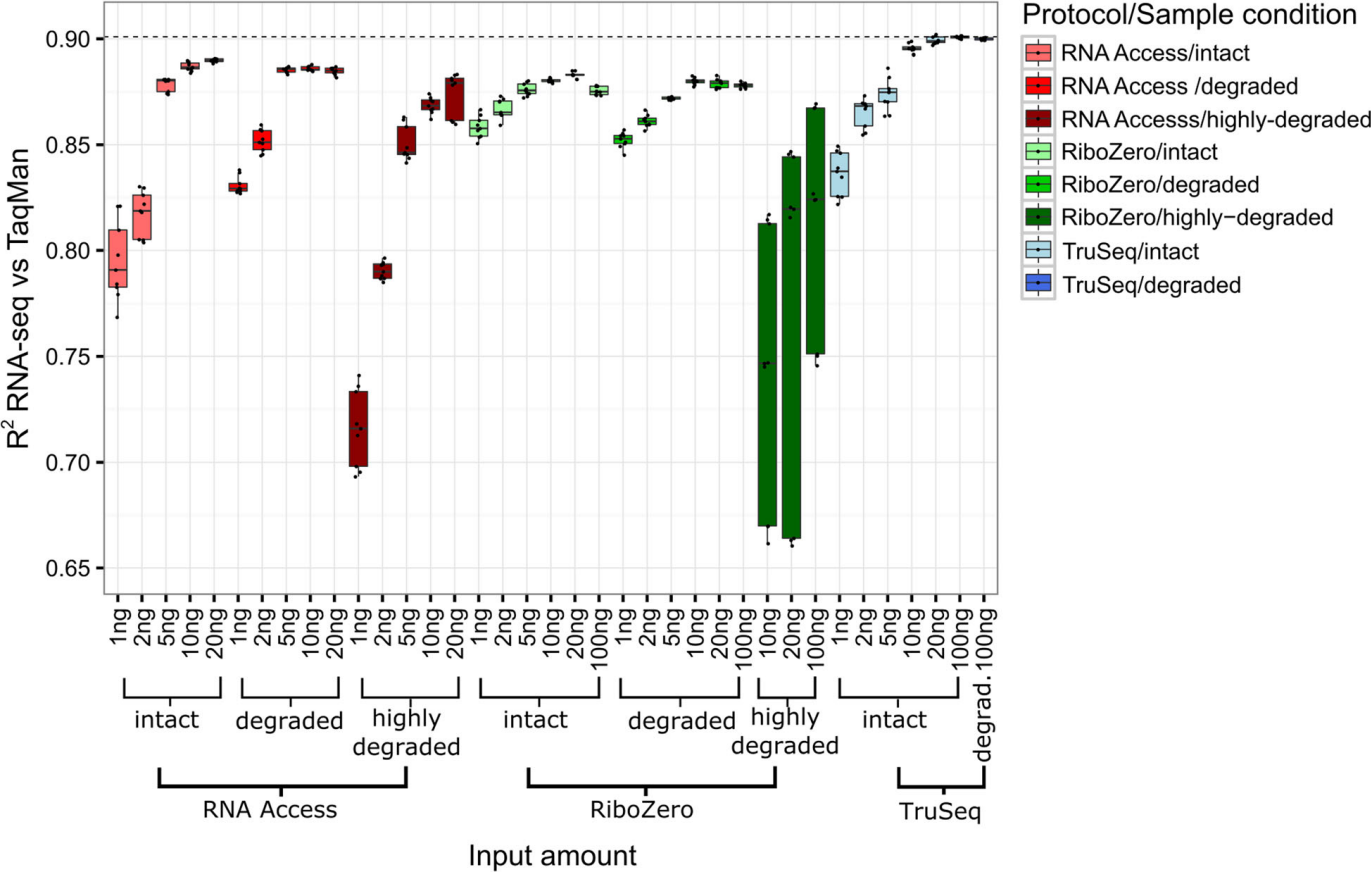
Boxplot of the coefficients of determination (*R* ^2^ values) of the RNA-seq log fold change values vs TaqMan qPCR measurements. The boxes are coloured by protocol: red for RNA Access, green for Ribo-Zero, and blue for TruSeq. Darker shades indicate boxes for samples to which a more severe degradation protocol was applied

We performed pairwise comparisons of log fold changes between the individual technical replicates and the TaqMan qPCR fold change values (Fig. 6). At recommended RNA input levels and on intact samples, TruSeq correlated better with the TaqMan qPCR data than the other two protocols (mean *R* ^2^ value of 0.9 vs 0.89 for RNA Access and 0.88 for Ribo-Zero). The concordance with qPCR fold changes decreased consistently with lower input amounts for all three protocols on intact samples. The log fold change correlation for the RNA Access protocol remained stable until the low input amount of 5 ng and then dropped considerably at 2 ng (from 0.88 to 0.82). A similar behaviour was observed for TruSeq where the log fold change correlation was high at input amounts of 100 ng and 20 ng (0.89–0.9) and then dropped to 0.87 at 5 ng and to 0.84 at 1 ng. Instead, Ribo-Zero showed only a slight decrease in the correlation of log fold changes down to the lowest input amount of intact RNA (R^2^ = 0.86 at 1 ng).

The correlation profiles for degraded sample inputs were similar to the intact RNA profiles (Fig. 6). TruSeq had the highest agreement with TaqMan at the recommended input amount (100 ng for TruSeq and Ribo-Zero and 20 ng of degraded RNA for RNA Access); *R* ^2^ values for the RNA Access protocol again declined sharply at inputs of 2 ng and 1 ng, while similar to intact sample inputs the *R* ^2^ values for Ribo-Zero went down gradually with decreasing input amount, being the best performing protocol at the lowest input amount (R^2^ = 0.85 at 1 ng).

Finally, when considering highly degraded RNA input, we observed a marked difference between the RNA Access and the Ribo-Zero protocols where the latter displayed a very high variability and lower median *R* ^2^ values at all tested inputs (down to 10 ng). The RNA Access approach, in comparison, performed much better on the highly degraded material. While the median *R* ^2^ values decreased from 0.87 for 20 ng down to 0.85 for 5 ng and dropped further at 2 ng and 1 ng to an *R* ^2^ value of 0.79, we found much less variability among the value distribution as compared to the Ribo-Zero method.

Overall our data suggest that all three protocols are robust and comparable on intact RNA samples and down to 10 ng of input. Going further down with the amounts, Ribo-Zero outperforms the other two protocols at 1 ng. In addition Ribo-Zero performs equally well on degraded RNA, closely followed by the RNA Access method. On severely degraded samples, where the RNA fragments are shorter than 200 nucleotides, Ribo-Zero reaches its limits and becomes much less reproducible. Here the RNA Access approach represents the best choice, still generating reliable data down to 5 ng input.

### Agreement between protocols

**Fig. 7:**
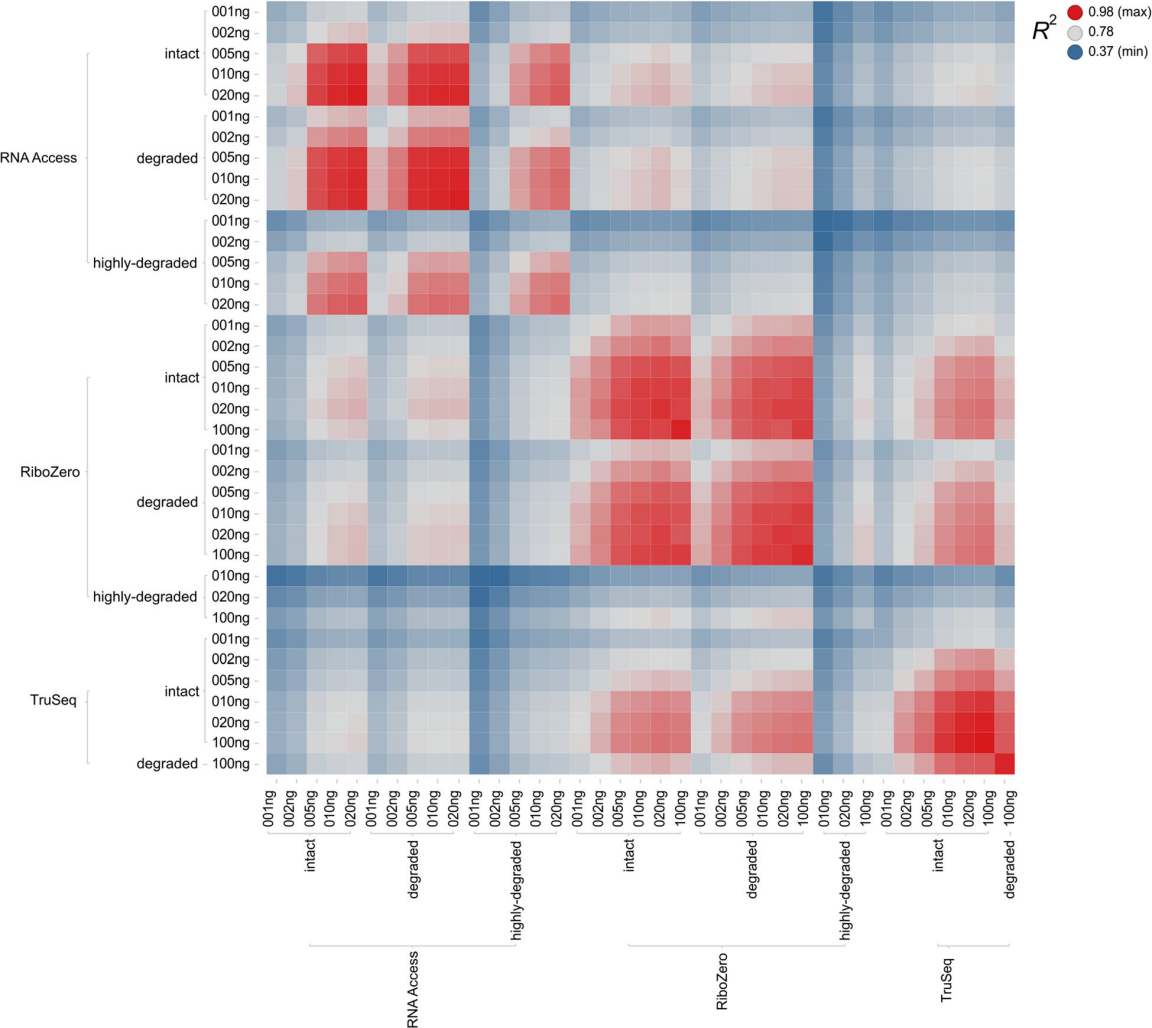
Heat map of the coefficients of determination (*R* ^2^ values) of the log fold change values of the pairwise comparison of all protocols. Each colored box represents the coefficient of determination (*R* ^2^ value) between two conditions which are given by the labels on the x and y axes. The *R* ^2^ value is color-coded on a scale where blue represents the lowest, grey the median, and red the highest observed value

To assess the similarity between the three protocols we computed the coefficients of determination (*R* ^2^ values) of the log fold changes of the SEQC samples for every combination of protocol, sample quality, and input amount (Fig. 7). For each condition, we first calculated the log fold changes and the corresponding *R* ^2^ values of each of three technical replicates separately. We then averaged the individual *R* ^2^ values of the three technical replicates to calculate the mean *R* ^2^ values reported in Fig. 7.

Overall we found that the similarity between samples increases with the input amount, and independently of the protocol and the sample quality. We then assessed the sample similarity within each protocol separately. For RNA Access the results on intact samples for 20 ng, 10 ng and 5 ng are highly similar (with *R* ^2^ values ranging from 0.97 to 0.9) whereas the similarity drops significantly at 2 ng (0.68) and 1 ng (0.61). These results are similar to those observed for the correlation with the TaqMan qPCR data. There was almost no difference between the intact and degraded samples resulting in *R* ^2^ values from 0.92 to 0.97 (for input amounts of at least 5 ng) – independent of which combinations were considered; however, there was a significant drop for the highly degraded samples with *R* ^2^ values ranging from 0.79 to 0.89 (again for input amounts of at least 5 ng).

Among Ribo-Zero samples the similarity values were also very high (with *R* ^2^ values above 0.91) for input amounts of at least 5 ng but the decrease in self-similarity to 2 ng (0.83) and 1 ng (0.76) was not as drastic as for RNA Access. Again, the *R* ^2^ values of the intact and degraded samples were essentially interchangeable with respect to other Ribo-Zero samples as well as other protocols. But for the highly degraded samples only the log fold changes of the input amount 100 ng showed some agreement with the higher input amounts (100 ng and 20 ng) of the intact and degraded Ribo-Zero samples (with *R* ^2^ values ranging from 0.77 to 0.8). In terms of self-similarity the achieved *R* ^2^ values (0.41 for 10 ng, 0.53 for 20 ng, and 0.69 for 100 ng) were comparable to the self-similarity *R* ^2^ values of the RNA Access protocols for input amounts that were an order of magnitude lower (0.46 for 1 ng, 0.59 for 2 ng, and 0.79 for 5 ng).

For TruSeq we found a very good agreement of the log fold change values for input amounts of 100 ng down to 10 ng (with *R* ^2^ values between 0.98 and 0.92) and then a significant drop for 5 ng and lower with self-similarity *R* ^2^ values from 0.82 down 0.61. Similar to the comparison with the TaqMan qPCR data, we again found an excellent agreement between the degraded and the intact samples despite the 3′ enrichment that we observed for the degraded sample.

When comparing the different protocols between each other, Ribo-Zero and TruSeq were more similar to each other than to RNA Access with the *R* ^2^ values ranging from 0.85 to 0.88 for intact samples and input amounts of 10 ng and above. For the same input amounts, RNA Access achieved only *R* ^2^ values of 0.8 to 0.82 with Ribo-Zero and 0.74 to 0.76 for TruSeq.

Overall the reproducibility of the three protocols was very good even for small input amounts down to 5 ng but the gene expression values exhibited considerable protocol specific biases leading to a reduced agreement between the different protocols. In general, the expression values generated by TruSeq and Ribo-Zero were more similar to each other than they were to those from RNA Access.

## Discussion

With the substantial reduction in the cost of sequencing, RNA-seq has fast become more affordable and gained popularity as major tool for research and potential clinical applications in medicine [23, 35]. While many studies have demonstrated its applicability for the discovery of disease-specific markers and therapeutic targets (16–22), new efforts are underway to expand this technology into clinical practice towards the establishment of RNA-seq-based clinical gene tests in regulated environments [23]. The wealth of valuable gene expression knowledge that can be gained with RNA-seq is however critically dependent on the quality and amount of the samples employed in the study.

When planning a gene expression profiling study by RNA-seq, the investigator is usually confronted with several decisions about the experimental design including the choice of the sequencing protocol. Several commercial protocols can be used depending on the quantity and quality of the RNA samples. In some cases, for instance when working with precious patient samples, it may be difficult to obtain enough RNA amount or material of sufficient quality to meet the vendor criteria for using standard kits such as the Illumina poly(A)-based TruSeq Stranded mRNA Kit (TruSeq) or the Illumina Ribo-Zero rRNA Removal Kit (Ribo-Zero). If the RNA quantity available for the experiment is significantly below the recommended starting amount of these kits (100 ng total RNA), amplification methods like the NuGEN Ovation or the Clontech SMARTer can be applied to picogram amounts of the RNA samples. This extra amplification, however, invariably incorporates additional biases to those normally introduced by the library construction and sequencing procedures, and hence should be avoided if possible. On the other hand, if the RNA quality is low, the Ribo-Zero kit and the new Illumina RNA Access Kit (RNA Access) may represent valid options for the study but have not been comprehensively evaluated for accuracy and performance on low quantity and low quality RNA thus far.

In this study, we performed a comprehensive and systematic assessment of two of the most commonly used commercial sequencing kits TruSeq and Ribo-Zero as well as the relatively new RNA Access kit covering a wide range of input amounts from 100 ng down to 1 ng and three sample quality stages from intact to highly-degraded. This input range is one of the most difficult to work with since the RNA amount is mostly below the sample requirements of the TruSeq and Ribo-Zero protocols, but still in an area where it would be ideal to avoid RNA amplification. We further aimed at understanding how the performance of these three protocols changes when the quality of the samples is not optimal or even heterogeneous across the RNA sample population. A summary of the results of the protocol assessment is presented in Table 1.

**Table 1:** Summary of the assessment of the three protocols for different input amounts and sample degradation stages

RNA seq Protocol	RNA Input	1 ng	2 ng	5 ng	10 ng	20 ng	100 ng	Conclusion
TruSeq stranded mRNA	Intact	=	+	++	+++	+++	+++	+Works well at low amounts down to 5 ng -Captures polyadenylated RNAs only -Not suited for degraded or highly degraded samples
	Degraded	*	*	*	*	*	++	
	Highly deg.	*	*	*	*	*	*	
RiboZero stranded RNA	Intact	+	+	++	++	++	++	+Works well over all input amounts +Captures all RNAs (coding & non coding) +Compares well to mRNA protocol +Well suited for degraded samples -Requires higher sequencing depth -Not suited for highly degraded samples
	Degraded	+	+	++	++	++	++	
	Highly deg.	*	*	*	−−	−	−	
RNA access	Intact	−	=	++	++	++	*	+Performs well on all samples down to 5 ng +Requires less sequencing depth +Suited for degraded and highlydegraded samples -Captures only preselected RNAs and is only available for human samples -Less similar to the other two protocols
	Degraded	−	=	++	++	++	*	
	Highly deg.	−−	−	=	+	+	*	

A +, ++, or +++ indicates that the protocol performed (very) well on the input, = indicates borderline performance, and a – or – – indicates an unsatisfactory performance. The symbol * is used to indicate input conditions that were not tested

As already noted in previous studies [25, 27, 30], in the ideal situation with enough input material and good sample quality, the choice of the sequencing protocol depends largely on the questions addressed by the study. For the input amounts recommended by the manufacturer, if the focus of the analysis is mainly on protein coding genes, then TruSeq is the protocol of choice due to its high alignment rate against exons (Fig. 2 and Additional file 1: Figure S4) and slightly better concordance with TaqMan qPCR data (Fig. 6). If other RNA species are also of interest, then Ribo-Zero is preferable as it can detect the largest transcript population capturing all coding and non-coding RNAs (Fig. 4). However, the Ribo-Zero protocol requires a higher sequencing depth as about 30% of the reads align against introns (Fig. 2 and Additional file 1: Figure S4) and are, thus, non-informative for the quantification of gene expression. This may have a small or large impact on the cost of the sequencing experiment depending on the sample size of the study. For low quantity inputs and down to 5 ng, all three tested library preparation protocols performed similarly well on intact RNA, despite being at much lower input quantities than the recommended amounts. At very low input amounts such as 1 ng and 2 ng, Ribo-Zero showed clear performance advantages over the other two protocols and still provides accurate gene expression change levels (Fig. 6).

If we consider degraded samples, then according to *Adiconis* et al. [25], the best RNA sequencing protocol for degraded samples was RNAse H in 2013. This protocol is a custom solution and not necessarily suitable for all laboratories or clinical centres. The commercial Illumina Ribo-Zero approach performed almost as well as RNAse H on degraded samples in their hands. We herein confirm that the Illumina Ribo-Zero ribosomal RNA depletion kit is a robust approach that worked well on very low input amounts down to 1 ng as well as on degraded samples (Fig. 6). However this approach did not perform well on highly degraded samples where the mean size of the library fragments is shorter than 200 nucleotides (Fig. 6). These short degraded RNA fragments tend to be removed during the library preparation steps, introducing a bias for accurate quantification.

The capture based approach of the RNA Access kit, which enriches for reads mapping to exons performed very well even on severely degraded samples and at medium to low input amounts down to 5 ng (Fig. 6). Moreover, this kit showed the most consistent mapping rates across the wide range of input amounts and quality levels tested (Fig. 2 and Additional file 1: Figure S4). Because of the RNA exome enrichment step, this protocol requires lower sequencing depth per sample than Ribo-Zero to generate high-quality data in high-value content regions. It therefore represents a more cost-effective solution if the cost of the enrichment kit is distributed across a large sample set.

## Conclusions

In summary, we conclude that RNA Access represents an attractive alternative for those studies in which the sample quality is severely compromised. For its broad application across the entire sample set, this protocol is also suited for the profiling of very heterogeneous RNA sample populations covering a wider range of low quantity and extremely low quality samples thus ensuring high accuracy and comparability of the results within the study.

## Methods

### Samples

SEQC samples A and B were prepared by adding ERCC [36] spike-in to two reference RNAs and as described in the Sequencing Quality Control study (SEQC) [34]. In brief, Universal Human Reference RNA (UHRR, #740000, Agilent Technologies) at 1 μg/μl was supplemented with 2% of ERCC ExFold RNA Spike-in Control Mix (ERCC1, #4456739, Life Technologies) to give Sample A. Human Brain Reference RNA (HBRR, # AM6050, Life TEchnologies) at 1 μg/μl was supplemented with 2% of ERCC ExFold RNA Spike-in Control mix 2 (ERCC2, #4456739, Life Technologies) to give Sample B. Bioanalyzer profiles of the intact SEQC samples are shown in Additional file 1: Figure S1.

To obtain degraded RNA, aliquots of SEQC samples A and B were incubated at 94 °C for 30 min (degraded samples-size peak at ~800 bp) and for either 60 min (highly degraded Sample A-size peak ~200 bp) or 210 min (highly degraded Sample B-size peak <200 bp). Bioanalyzer profiles of the samples before and after degradation are shown in Additional file 1: Figures S2 and S3. Intact RNA displays the characteristic fragment size peaks for 18S and 28S rRNA whereas these peaks vanish for degraded and highly degraded RNA.

### Sequencing libraries

Poly-A enriched strand-specific libraries were generated with the TruSeq mRNA V2 sample preparation kit (#RS-122-2001, Illumina), ribosomal RNA depleted strand-specific RNA libraries with the TruSeq Stranded Total RNA LT sample preparation kit with Ribo-Zero Gold (#RS-122-2301and (#RS-122-2302, Illumina), and transcriptome capture based libraries with the TruSeq RNA Access Library Prep Kit (#RS-301-2001, Illumina). All protocols were performed following the manufacturer’s instructions. Recommended amounts of starting material were as follows: 100 ng of input RNA for TruSeq, 100 ng for Ribo-Zero, and 10 ng of intact RNA or 20 ng of degraded RNA for RNA access.

Libraries were generated for different input amounts of total RNA (1 ng, 2 ng, 5 ng, 10 ng, 20 ng and 100 ng) in triplicates. Ribosomal RNA depleted RNA was fragmented for 8 min (intact and degraded samples) or 2 min (highly degraded samples), different fragmentation times. qPCR was performed on unamplified libraries to evaluate the appropriate amplification cycles number of the large-scale PCR step in library preparation. The quality and yield of the prepared libraries was assessed using an Agilent 2100 Bioanalyzer (Agilent Technologies, Santa Clara, CA, USA).

### Sequencing

Sequencing was performed on a HiSeq2500 Instrument (Illumina) with 2 × 76 cycles, using either the Illumina TruSeq v3 or TruSeq Rapid SBS sequencing chemistry and following the manufacturer’s instructions. Images from the instrument were processed using the manufacturer’s software to generate FASTQ sequence files. Read quality was assessed by running FastQC (version 0.10) on the FASTQ files. Raw RNA-sequencing reads were deposited in the NCBI Short Read Archive under the accession number SRP097611.

### Alignment and quantification

We used the Exon Quantification Pipeline 2.0 [37] to align the reads against the human genome reference files from Ensembl version 76 [38] and quantify gene expression. For computing the 5′ to 3′ coverage along transcripts we used the Picard tool CollectRnaSeqMetrics version 1.86 [39]. For each sample, gene counts were divided by the total number of mapped reads and multiplied by one million to obtain Counts Per Million (CPMs) to account for varying library sizes. Fragments per kilobase per million mapped reads (FPKM) were calculated by dividing the CPM values by the gene lengths.

For the computation of fold changes (FC) of CPM values a pseudo count of 0.5 CPM was added to both values. The computation of the number of expressed genes was based on a FPKM cut-off of 0.3 [40] and the set of genes detected in both samples (SEQC-A and SEQC-B) were combined. Gene categories were based on the Ensembl “Gene Type” field for which we aggregated different subcategories to obtain a more coarse-grained better interpretable result (Additional file 1: Table S1).

For the computation of the coefficients of determination (*R* ^2^) of the log fold change values, we first calculated the log fold changes and the corresponding *R* ^2^ values of each of three technical replicates separately. We then averaged the individual *R* ^2^ values of the three technical replicates to calculate the mean *R* ^2^ values for each specific combination of input amount, degradation stage, and protocol.

### TaqMan data

The TaqMan qRT-PCR data for the SEQC-A and B samples were downloaded from Gene Expression Omnibus under the accession number GSE5350.

## Additional file

Additional file 1: Figure S1. Bioanalyzer profile of the fragment size distribution for the intact SEQC-A and SEQC-B samples. The curve for SEQC-A is shown in red and the curve for SEQC-B in blue. The two peaks represent the intact 18S and 28S ribosomal RNA profiles. **Figure S2.** Bioanalyzer profile of the fragment size distribution for the degraded SEQC-A and SEQC-B samples. The curve for SEQC-A is shown in red and the curve for SEQC-B in blue. The peaks for the 18S and 28S ribosomal RNAs are now following a unimodal distribution with a much wider peak around a fragment size of 850 nt, reflecting the level of degradation. **Figure S3.** Bioanalyzer profile of the fragment size distribution for the highly-degraded SEQC-A and SEQC-B samples. The curve for SEQC-A is shown in red and the curve for SEQC-B in blue. The peaks for the 18S and 28S ribosomal RNAs are now following a unimodal distribution with a much wider peak around a fragment size of 150–200 nt, reflecting a high level of degradation. **Figure S4.** Bargraph of the alignment statistics for the SEQC-B sample and all three protocols. Each bar represents the averaged values across the three technical replicates per condition. The percentage of total aligned reads is represented by the height of the bar, and the percentage of reads aligning to exons is in red, introns in blue, and intergenic regions in green. **Figure S5.** Venn diagram of the protein coding genes detected by each of the three protocols. Venn diagram of the protein coding genes detected by each of the three protocols on degraded samples at the input amounts 10 ng for RNA Access and 100 ng for Ribo-Zero and TruSeq. A gene is considered “expressed” if it has a FPKM value of at least 0.3 in one of the three technical replicates of at least one of the two samples (SEQC-A or SEQC-B). **Table S1.** Simplified Ensembl gene type mapping. The original Ensembl (v76) gene type category is contained in the left column and the simplified category is contained in the right column. (PDF 661 kb)

## Abbreviation

MAQC/SEQC: Microarray/Sequencing Quality Control
