# Supplementary material for: A comprehensive assessment of RNA-seq protocols for degraded and low-quantity samples
Source: BMC Genomics. 2017 Jun 5;18:442. doi: 10.1186/s12864-017-3827-y (PMC5460543; doi:10.1186/s12864-017-3827-y)
Supplement: Additional file 1: Figure S1. — Bioanalyzer profile of the fragment size distribution for the intact SEQC-A and SEQC-B samples. The curve for SEQC-A is shown in red and the curve for SEQC-B in blue. The two peaks represent the intact 18S and 28S ribosomal RNA profiles. Figure S2. Bioanalyzer profile of the fragment size distribution for the degraded SEQC-A and SEQC-B samples. The curve for SEQC-A is shown in red and the curve for SEQC-B in blue. The peaks for the 18S and 28S ribosomal RNAs are now following a unimodal distribution with a much wider peak around a fragment size of 850 nt, reflecting the level of degradation. Figure S3. Bioanalyzer profile of the fragment size distribution for the highly-degraded SEQC-A and SEQC-B samples. The curve for SEQC-A is shown in red and the curve for SEQC-B in blue. The peaks for the 18S and 28S ribosomal RNAs are now following a unimodal distribution with a much wider peak around a fragment size of 150–200 nt, reflecting a high level of degradation. Figure S4. Bargraph of the alignment statistics for the SEQC-B sample and all three protocols. Each bar represents the averaged values across the three technical replicates per condition. The percentage of total aligned reads is represented by the height of the bar, and the percentage of reads aligning to exons is in red, introns in blue, and intergenic regions in green. Figure S5. Venn diagram of the protein coding genes detected by each of the three protocols. Venn diagram of the protein coding genes detected by each of the three protocols on degraded samples at the input amounts 10 ng for RNA Access and 100 ng for Ribo-Zero and TruSeq. A gene is considered “expressed” if it has a FPKM value of at least 0.3 in one of the three technical replicates of at least one of the two samples (SEQC-A or SEQC-B). Table S1. Simplified Ensembl gene type mapping. The original Ensembl (v76) gene type category is contained in the left column and the simplified category is contained in the right column. (PDF 661 kb) [file 12864_2017_3827_MOESM1_ESM.pdf]

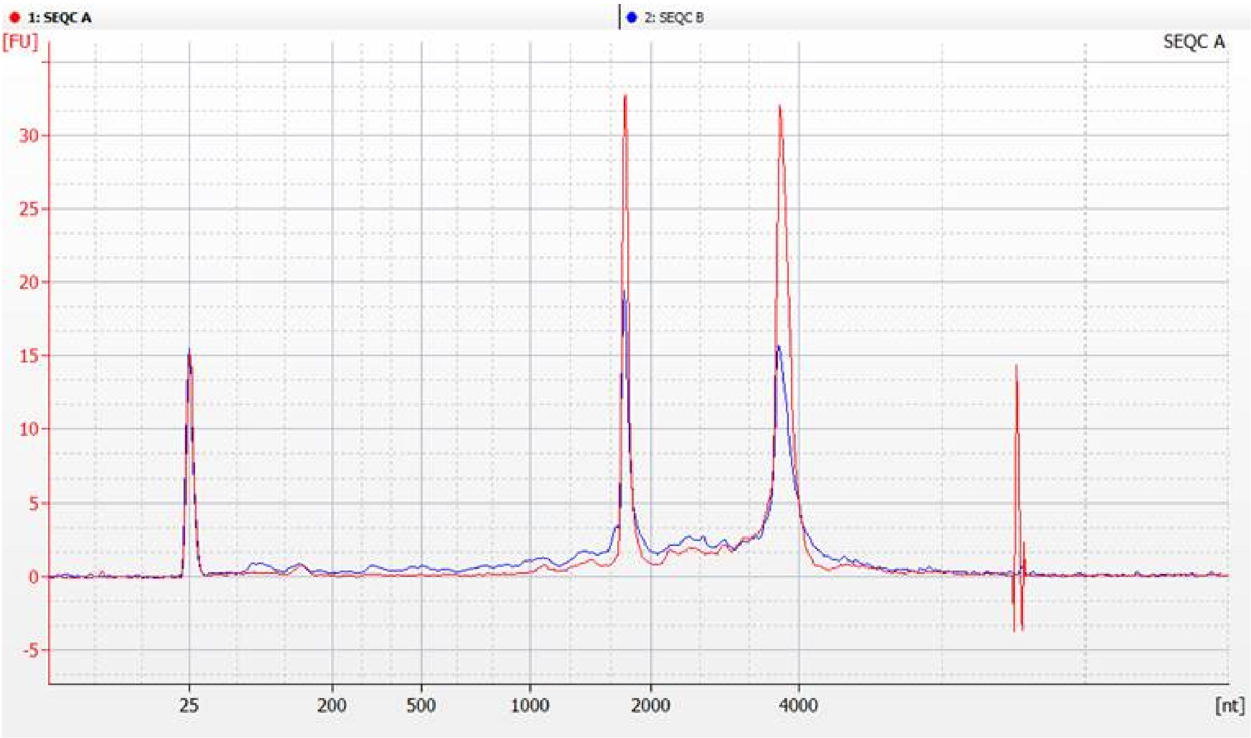

Figure S1: Bioanalyzer profile of the fragment size distribution for the intact SEQC-A and SEQC-B samples. The curve for SEQC-A is shown in red and the curve for SEQC-B in blue. The two peaks represent the intact 18S and 28S ribosomal RNA profiles.

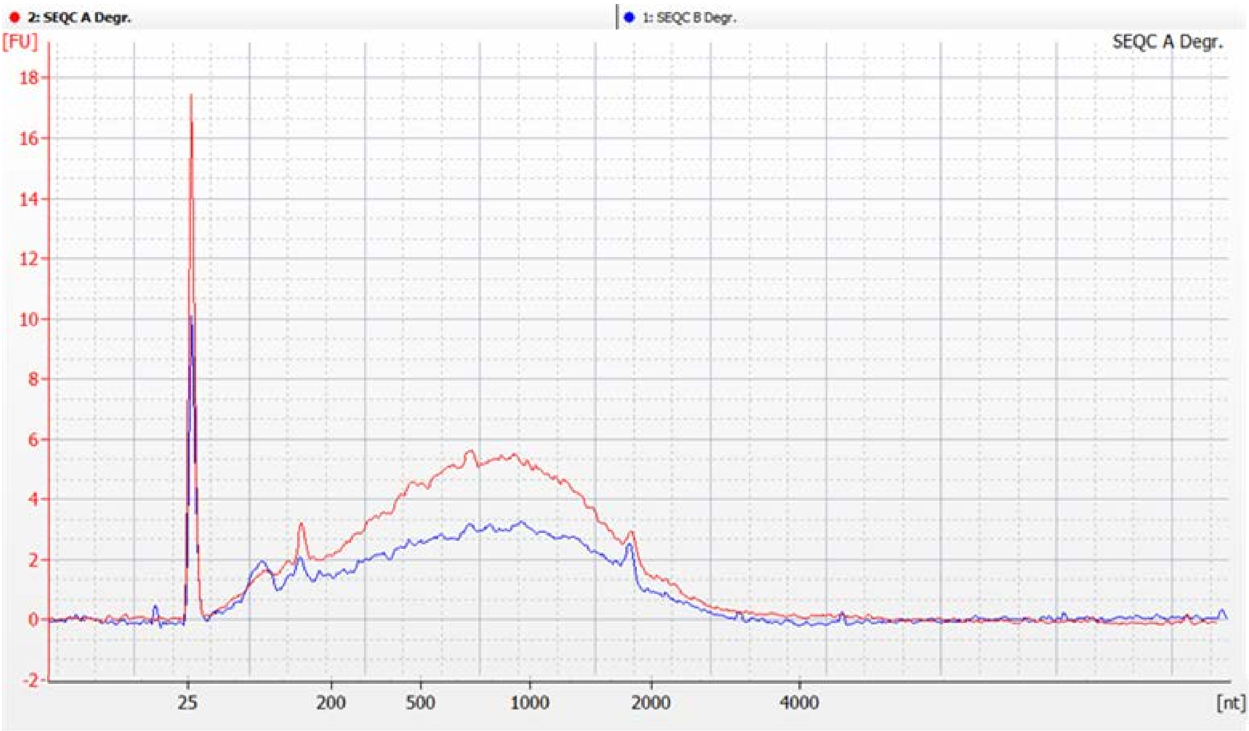

Figure S2: Bioanalyzer profile of the fragment size distribution for the degraded SEQC-A and SEQC-B samples. The curve for SEQC-A is shown in red and the curve for SEQC-B in blue. The peaks for the 18S and 28S ribosomal RNAs are now following a unimodal distribution with a much wider peak around a fragment size of 850 nt, reflecting the level of degradation.

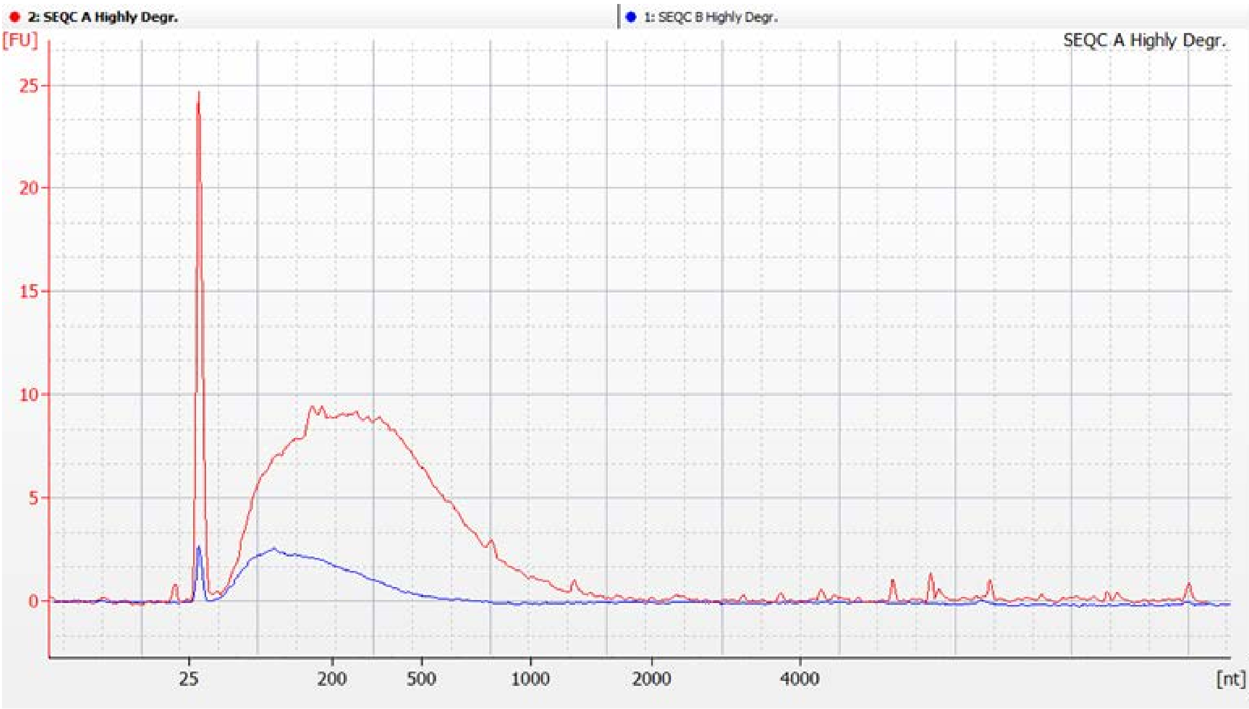

Figure S3: Bioanalyzer profile of the fragment size distribution for the highly-degraded SEQC-A and SEQC-B samples. The curve for SEQC-A is shown in red and the curve for SEQC-B in blue. The peaks for the 18S and 28S ribosomal RNAs are now following a unimodal distribution with a much wider peak around a fragment size of 150-200 nt, reflecting a high level of degradation.

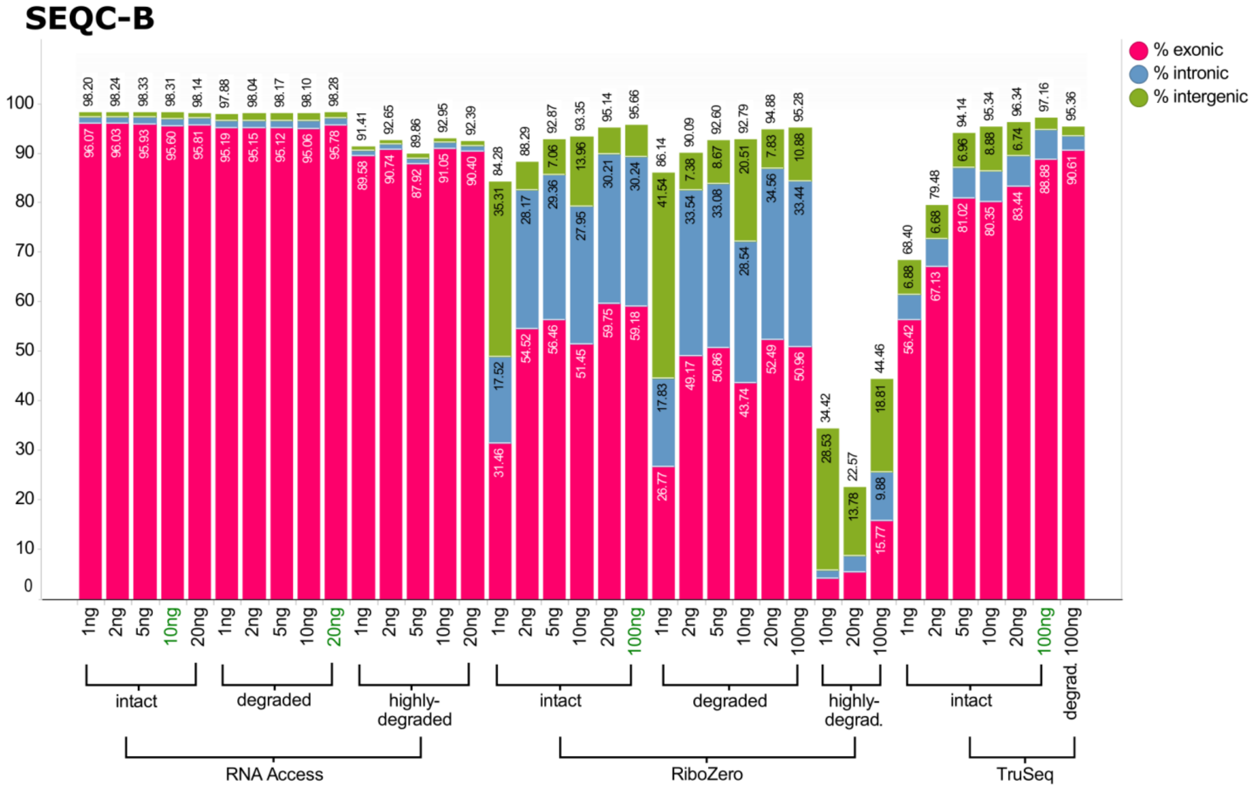

Figure S4: Bargraph of the alignment statistics for the SEQC-B sample and all three protocols. Each bar represents the averaged values across the three technical replicates per condition. The percentage of total aligned reads is represented by the height of the bar, and the percentage of reads aligning to exons is in red, introns in blue, and intergenic regions in green.

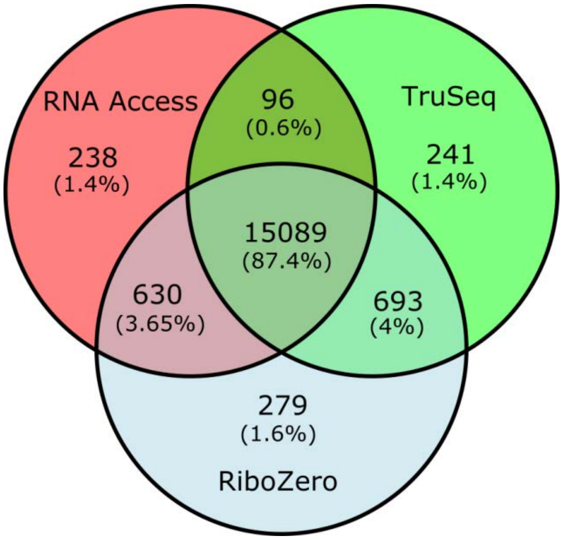

Figure S5: Venn diagram of the protein coding genes detected by each of the three protocols on degraded samples at the input amounts 10 ng for RNA Access and 100 ng for Ribo-Zero and TruSeq. A gene is considered “expressed” if it has a FPKM value of at least 0.3 in one of the three technical replicates of at least one of the two samples (SEQC-A or SEQC-B).

Ensembl biotype	Consolidated biotype
miRNA	smallRNA
piRNA	smallRNA
snRNA	smallRNA
snoRNA	smallRNA
protein_coding	Protein coding
rRNA	rRNA
Mt_rRNA	rRNA
Mt_tRNA	tRNA
lincRNA	lncRNA
antisense	lncRNA
sense_intronic	lncRNA
sense_overlapping	lncRNA
3prime_overlapping_ncrna	lncRNA
IG_C_gene	Immunoglobulin gene
IG_D_gene	Immunoglobulin gene
IG_J_gene	Immunoglobulin gene
IG_V_gene	Immunoglobulin gene
TR_C_gene	Immunoglobulin gene
TR_J_gene	Immunoglobulin gene
TR_V_gene	Immunoglobulin gene
TR_D_gene	Immunoglobulin gene
pseudogene	pseudogene

IG_C_pseudogene	pseudogene
IG_J_pseudogene	pseudogene
IG_V_pseudogene	pseudogene
TR_J_pseudogene	pseudogene
TR_V_pseudogene	pseudogene
Mt_tRNA_pseudogene	tRNA
tRNA_pseudogene	tRNA
snoRNA_pseudogene	pseudogene
snRNA_pseudogene	pseudogene
scRNA_pseudogene	pseudogene
rRNA_pseudogene	rRNA
misc_RNA_pseudogene	pseudogene
miRNA_pseudogene	pseudogene
transcribed_processed_pseudogene	pseudogene
transcribed_unprocessed_pseudogene	pseudogene
translated_processed_pseudogene	pseudogene
unitary_pseudogene	pseudogene
unprocessed_pseudogene	pseudogene
polymorphic_pseudogene	pseudogene
processed_pseudogene	pseudogene
processed_transcript	pseudogene
LRG_gene	misc RNA
misc_RNA	misc RNA

Table S1: Simplified Ensembl gene type mapping. The original Ensembl (v76) gene type category is contained in the left column and the simplified category is contained in the right column.
